# Editor's Letter-Box

**Published:** 1899-04-01

**Authors:** 


### Editor's Letter-Box.

[Our Correspondents are reminded that prolixity is a great bar to publication, and that brevity of style and conciseness of statement greatly facilitate early insertion.]

Mr. C. J. S. Thompson writes: In your kind remarks on my book, "Poison Romance and Poison Mysteries," in your issue of the 18th inst., you ask, is *Bish* the distinctive Hindustani name for aconite? In the "Pharmacographia" of Flückiger and Hanbury, which is generally regarded as an authority among pharmacologists, *Bish*, *Bis*, or *Bikh* are given as synonyms for Indian or Nepal aconite. You will also find the following statements:—"The poisonous root known in India as *Bish*, *Bis*, or *Bikh* is chiefly derived from Aconitum ferox. The roots of this and other species are collected indiscriminately according to Hooker and Thomson, under the name of *Bish* or *Bikh*." *Bish* is the name used by the Persian physician Alherir, as well as by Avicenna. The Arabic name *Bish* or Persian *Bis* is stated by Mordean Sheriff to be a more correct designation than *Bikh*. The Arabian writer, Ibu Buytur, also gives the word as *Bish*. I think there can be little doubt that the word *Bish* is generally used in India to designate Indian or Nepal aconite.

